# The role of body mass in limiting post heat‐coma recovery ability in terrestrial ectotherms

**DOI:** 10.1002/ece3.10218

**Published:** 2023-06-23

**Authors:** Chi Man Leong, Tin Yan Hui, Benoit Guénard

**Affiliations:** ^1^ School of Biological Sciences The University of Hong Kong Hong Kong SAR China; ^2^ The Swire Institute of Marine Science The University of Hong Kong Hong Kong SAR China; ^3^ Present address: Environmental Science Programme, Department of Life Sciences, Faculty of Science and Technology Beijing Normal University‐Hong Kong Baptist University United International College Zhuhai China; ^4^ Present address: Science Unit Lingnan University Hong Kong SAR China

**Keywords:** ecophysiology, fitness, heat shock, recovery, thermal resilience

## Abstract

Under global warming, animal species show shrinking body size responses, cascading deep changes in community structure and ecosystem functions. Although the exact physiological mechanisms behind this phenomenon remain unsolved, smaller individuals may benefit from warming climate more than larger ones. Heat‐coma, a physiological state with severe consequences on locomotion ability, is often considered as an “ecological death” scenario under which individuals are unable to escape and exposed to predation, further heat injury, and other hazards. Species are expected to increasingly encounter heat‐coma temperature thresholds under warming climate, and body size may be an important trait for thermoregulation in particular for ectotherms. The relationship between heat‐coma and shrinking body size remains, however, unclear. Yet, recovery after short‐term heat‐coma is possible, but little is known about its importance in thermal adaptation and how organismal size correlates with post heat‐coma recovery. Here, using ants as a model system, we firstly examined the fate of heat‐comatose individuals under field conditions to quantify the ecological benefits of post heat‐coma recovery. Then, we quantified ants' recovery ability after heat‐coma using a dynamic thermal assay in the laboratory and asked if thermal resilience varies between species with different body mass. Our results confirm that heat‐coma represents an inherent ecological death where individuals failed to recover from coma suffer strong predation pressure. Additionally, following phylogenetic signals inclusion, organisms with small mass were more likely to recover, supporting the temperature‐size rule in thermal adaptation and recent studies showing a decrease in body size composition of ectotherm community under warmer climatic conditions. Body size as a fundamental trait in ecology thus affects ectotherm survival under thermal stress, which may drive species body size adaptations and community composition under future warming scenarios.

## Graphical abstract

Under global warming, animal species show shrinking body size responses to heat. We found that heat‐comatose ants were suffering high predation pressure and thus were ecologically dead. Ants with small mass, however, were more thermal resilient and likely to recover from heat‐coma as compared to larger species, providing an explanation for the decrease in body size observed in ectotherms under warmer climatic conditions.

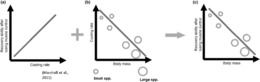

## 1 INTRODUCTION

Global warming has raised tremendous attention in the scientific community due to its impacts on biodiversity (García‐Robledo et al., 2016; Malcolm et al., 2006). Ectotherms, in particular, which have body temperatures that are strongly dependent on the ambient temperature, and which constitute the overwhelming majority of biodiversity, could be among the most vulnerable organisms to temperature increases (Angilletta et al., 2002; Pinsky et al., 2019). Already, global warming has been shown to impact both the abundance and diversity of ectotherms (González‐Tokman et al., 2020), potentially leading to cascading effects on pivotal ecosystem functions they provide in most ecosystems (Eggleton, 2020). Therefore, understanding the thermal resilience and sensitivity of ectotherms is urgently needed to predict how the performance and survival of ectotherms will be impacted under global change scenarios, and how to maintain ecosystems' health through well‐informed conservation decisions (Ma et al., 2020).

Among various predicted impacts of climate change, increased frequency of heat‐extreme events is likely to impact the fitness of ectotherms. From a biological standpoint, both thermal tolerance (e.g., the upper/lower thermal limit) and resilience (e.g., recovery ability after thermal coma) will, therefore, represent key physiological characteristics to evaluate ectotherm performance and persistence in the future (Johnson & Stahlschmidt, 2020; Kellermann & van Heerwaarden, 2019). Researchers use thermal performance curves, which model organismal performance as a function of body temperature, to understand how fitness and performances (e.g., growth, locomotion, metabolic rate) vary with body temperature (Angilletta Jr, 2009; Lutterschmidt & Hutchison, 1997b; Sinclair et al., 2016). Limited attention, however, has been paid on organism recovery responses when their body temperatures vary temporarily over the lower/upper thermal limits (Angilletta Jr, 2009; Marshall et al., 2011). A number of species can recover after momentary exposure to “lethal” heat stress (Furuki et al., 2017; Marshall et al., 2011), surviving through short‐term but extreme heat‐coma events, for example, heatwaves (Gaitán‐Espitia et al., 2013; Mori & Kimura, 2008). The consideration of recovery ability when assessing species responses to temperature changes is thus needed to avoid underestimation of individual survival, and to provide more realistic predictions of individual performance, population persistence, and community resilience under warming environments or heat‐extreme events.

Heat‐coma occurs when physiological thermoregulation fails and neural function shuts down (Jørgensen et al., 2020). As such, heat‐coma is considered a protective physiological mechanism to avoid further damages under thermal stress (González‐Tokman et al., 2020). Individuals in the heat‐coma state are often unable to move, though the synthesis of heat shock proteins may continue to chaperon vital metabolism under thermal stress (Gao et al., 2014; Hoffmann et al., 1997). Under natural conditions, however, entering heat‐coma is risky due to such immobility and thus the exposure to predators or scavengers (Wehner & Wehner, 2011). Thermal resilience can therefore be considered a crucial response to heat stress as much as thermal tolerance and should act as a thermal adaptative trait in response to warmer conditions.

In ectotherms, body mass represents one of the critical traits for thermal adaptation and physical heat exchange (Bergmann, 1848; Campbell & Norman, 1998), with thermal inertia playing a key role in both heat gain and loss which may ultimately affect recovery ability. The recovery ability after heat‐coma in ectotherms remains nonetheless unclear, particularly the relationship between heat‐coma recovery and body mass (e.g., Bozinovic et al., 2011; Willot et al., 2021). As extreme hot days and pulsed, short‐term heatwaves are becoming more frequent under climate change (Lee et al., 2021; Rahmstorf & Coumou, 2011), the recovery ability of organisms after extreme heat events could be a key trait for survival and act as a precursor to adapt to novel environmental conditions (Angilletta Jr, 2009).

**FIGURE 1:**
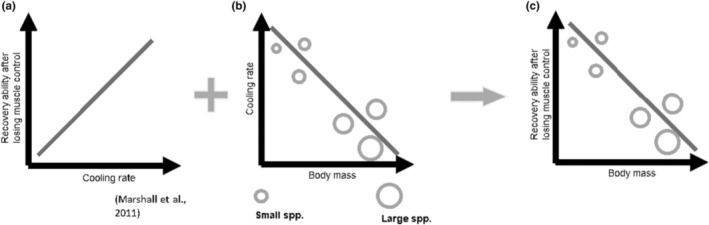
Schematic diagrams and hypotheses regarding the relationship between body mass and post heat‐coma recovery ability in ectotherms. (a) Firstly, individuals will recover faster from heat‐coma if their body temperatures can cool down at a faster rate from heat‐coma temperature (Bar‐Ziv & Scharf, 2018; Marshall et al., 2011). (b) Secondly, according to biophysical principles (Kühsel et al., 2017; Planinšič & Vollmer, 2008), smaller organisms have faster cooling rate than larger organisms due to higher surface area‐to‐volume ratio for heat exchange. (c) As such, the present study hypothesized that species with smaller mass have stronger recovery ability after heat‐coma.

Ants (Formicidae) have been widely used as an ectothermic model in thermal biology due to their biological and habitat diversity (Cerdá et al., 1998; Kaspari et al., 2015; Leong et al., 2022), and variations in body mass are often associated with the ecosystem functions they perform (Nooten et al., 2022; Séguin et al., 2014; Woodward et al., 2005). To understand the benefit of post heat‐coma recovery, we first validated the fate of heat‐comatose ant individuals in the field to test if heat‐coma condition is considered as “ecological death” (Angilletta Jr, 2009; Jørgensen et al., 2020). Using an in situ experiment, we tested the ecological importance of heat‐coma recovery as an adaptation to limit predation capture and validate the biological relevance of this trait. Then, we investigated ectotherms' recovery ability under thermal stress and tested whether such ability is correlated with body mass (Figure 1). To do so, we selected 29 ant species from eight subfamilies representing over three orders of magnitude in body mass (0.029–37.383 mg) to investigate the relationship between thermal resilience and body mass. Using dynamic thermal assays to simulate different heating regimes acting on the organisms (Leong et al., 2022; Lutterschmidt & Hutchison, 1997b), we quantified species thermal resilience and measured post heat‐coma recovery ability. Since body mass negatively correlates with the rate of heat exchange due to a higher surface area‐to‐volume ratio in smaller individuals (Planinšič & Vollmer, 2008), smaller species are expected to reach cooler body temperatures within a shorter period of time after heat stress as compared to larger species (Kühsel et al., 2017). We, thus, hypothesized that the recovery ability is expected to be higher in smaller compared to larger species.

## 2 MATERIALS AND METHODS

### 2.1 Field experiment for in situ predations on heat‐comatose ants

To determine the ecological relevance of recovery ability after heat‐coma, we conducted a predation experiment using heat‐comatose ants (i.e., alive ants were collected in the field and then heat‐shocked), and quantified predation rates on these comatose individuals in open urban and closed forest habitats across two sites in Hong Kong. The forest habitat consists of secondary forest, while the open urban habitat consists of a limited number of trees and is largely covered by grasslands. A total of 90 individuals representing six ant species of different body masses (species and their average dry weights: *Pheidole parva*: 0.04 mg, *Solenopsis invicta*: 0.18 mg, *Technomyrmex brunneus*: 0.23 mg, *Anoplolepis gracilipes*: 0.41 mg, *Leptogenys kitteli*: 1.58 mg, and *Oecophylla smaragdina*: 2.20 mg) were used in the experiment.

As the six species were present at different sites, only one of the six species was used each time for the predation experiment. The experiments were conducted only on hot days (i.e., with ambient air temperature > 33°C) to simulate the extremely hot conditions characterizing such events. In each experiment, a random transect of 80 m long was laid across the habitat and 15 white disks (Ø 4.7 cm) were placed along the transect (disks at least 5 m apart) for predation observation. Each individual was first transferred to an Eppendorf tube and then treated with hot water (60–65°C) for 1 min to induce long‐duration (>30 min) heat‐coma based on previous laboratory observations. Then, a single individual was placed on a white disk positioned on the ground surface for observations. During the observation period, we checked the status of the coma individuals every 5 min, and scored their status according to one of the following three categories: being captured, not being captured (present in their original location), or recovered.

### 2.2 Ant collection for heat‐coma recovery measurement

To quantify the thermal resilience of ants with distinct body sizes living in habitats with different thermal regimes, a total of 29 ant species from eight subfamilies were collected in monsoonal tropics in East Asia (32 sites from Hong Kong SAR and Macao SAR, China) during the dry season of 2018 and the wet seasons of 2018–2021. Ants were collected in both urban (nine species) and forest habitats (20 species) using hand collection (Brassard et al., 2021; Leong et al., 2017). We targeted the relatively abundant species present in these habitats as well as species representing a wide range of body sizes to reflect a gradient of body mass and thermal preferences (crymophilic or thermophilic; Lee et al., 2021). Only the worker caste was collected and assayed because workers tend to forage and scout efficiently during hot temperatures (Tross et al., 2021). Collected ants (ca. 120 individuals per species) were acclimated with wet cotton in the laboratory for over 2 h (Bujan & Kaspari, 2017; Leong et al., 2022) at The University of Hong Kong. They were then maintained in chambers lined with wet cotton at 24 ± 2°C for <3 days prior to thermal assays, in order to avoid thermal acclimation in laboratory conditions (García‐Robledo et al., 2016; Roeder et al., 2021).

### 2.3 Heat‐coma experiments and quantification of recovery ability

We used dynamic thermal assays (Leong et al., 2022) to induce heat‐coma in collected ants followed by recovery assessments to quantify the recovery ability of different species. To achieve that, each individual ant worker was taken from the acclimation chamber and placed in a 2.0 mL Eppendorf tube capped with cotton inside a digital dry bath (Benchmark BSH1004, stated accuracy ±0.2°C). The bath was set at 36°C, which is generally used as the starting point of the dynamic thermal assay in ants (Bujan et al., 2016). Based on microclimatic data of the foraging temperature conditions, we selected three ramping rates for the thermal assays (i.e., 0.2, 0.5, and 1.0°C/min); these three ramping rates are frequently applied as heat knockdown treatments for measuring critical thermal maximum in ants and are also considered environmental temperature relevant for ants (Leong et al., 2022). These were then used, in a randomized order among all 29 species, to simulate heat shock events of various rates. During each assay, a digital thermometer (UEi Test Instruments DT302) was placed inside an extra Eppendorf tube to measure the surface temperature inside the vials, which was used to represent the environmental temperature experienced by each individual (Kaspari et al., 2015).

Once an individual demonstrated signs of losing muscle control, a common phenomenon associated with heat‐coma in ectotherms (McMahon, 1976), it was transferred to room temperature, that is, 25°C, for 10 min to assess recovery. Such recovery temperature was pretested and compared between room temperature (25°C) and temperature of species heat‐coma minus 10°C (see Appendices S1 and S2; Table S1; and the field experiment below for adopting such conditions to assess recovery). Similar time period has also been adopted to assess recovery in *Drosophila* after heat‐coma events (Bozinovic et al., 2011). After 10 min, we assessed the status of each individual and recorded its locomotion ability (sensu Lutterschmidt & Hutchison, 1997a) to score for recovery failure (0) or success (1). After the recovery assay, each individual was briefly placed in a freezer and dried for 10 days at 40°C in a heat chamber (BINDER GmbH Model FD 23–20 L) and weighed using an ultra‐microbalance (Sartorius MCA3.6P‐2S00, accuracy: 0.001 mg) to determine individual dry weight. A total of 1826 specimens from eight subfamilies, 24 genera, and 29 species were tested for their recovery ability after heat‐coma.

### 2.4 Phylogenetic signals

In considering whether phylogenetic signals were present in the dry weight or recovery rate among the assayed species, dry weight and recovery rate (i.e., proportion of individuals which recovered) were averaged at the genus level and phylogenetic signal analysis performed on these genus‐averaged traits. Ant ecophysiology, habitat, and morphology are highly specific at the genus level (Hölldobler & Wilson, 1990; Lucky et al., 2013), and therefore, we used a backbone tree based on genus‐level phylogeny (Economo et al., 2018), and applied tree pruning to keep the genera present in our samples (24 genera) in generating a genus‐level phylogeny. We then calculated Pagel's *λ* and Blomberg's *K* for phylogenetic signals of post heat‐coma recovery using the package *phytool* (Revell, 2012).

### 2.5 Data analyses

To determine whether heat‐comatose individuals were suffering higher capture rates by predators under natural conditions when exposure time increased, we performed a linear mixed model with capture rate (continuous) as the response variable, exposure time (continuous), and habitat (factor with two levels) as the predictor variables using the *lme4* package. To control for species habitat preference, we specified species nested within the habitat as random intercepts in the model. Lastly, we used *MuMin* package and *r.squaredGLMM* function (Barton & Barton, 2015) to calculate marginal ($R_{m}^{2}$) and conditional *R* ^2^ ($R_{c}^{2}$) for each linear mixed model, which quantifies variances explained by fixed effects alone ($R_{m}^{2}$) and both fixed and random effects ($R_{c}^{2}$).

To determine whether recovery ability varied with ramping rate and body mass, we constructed a generalized linear model (GzLM) with species recovery ability as the response variable, average dry weight (continuous), ramping rate (a factor with three levels), subfamily (a factor with eight levels) and their interactions as potential predictor variables. While subfamily correlates with body mass of ants, there were overlaps in body size between subfamilies and, therefore, we included subfamily as one of the potential predictors. A beta error distribution was used for the GzLM as recovery rate bounds between 0 and 1. Recovery data having 0 or 1 values were transformed prior to fitting the GzLM (Cribari‐Neto & Zeileis, 2009). We used Akaike Information Criteria (AIC) to compare models with different interaction combinations among the three predictor variables, and the model with the lowest AIC was selected. To avoid the scale‐of‐choice effect, which may confound the model results when subfamilies were pooled in the analyses, we further performed the GzLM within each subfamily (Rolán‐Alvarez et al., 2015). No phylogenetic contrasts were included in the GzLM due to the absence of significant phylogenetic signals in recovery responses (see Section 3). The GzLM was performed using the *glm* package (Mueller & Seneviratne, 2012). The figures were generated using *ggplot2* (Wickham, 2016) and modified using Adobe illustrator CC 2015. All analyses were conducted using the R program 4.0.5 (R Development Core Team, 2020).

## 3 RESULTS

### 3.1 In situ predation on heat‐comatose ants

**FIGURE 2:**
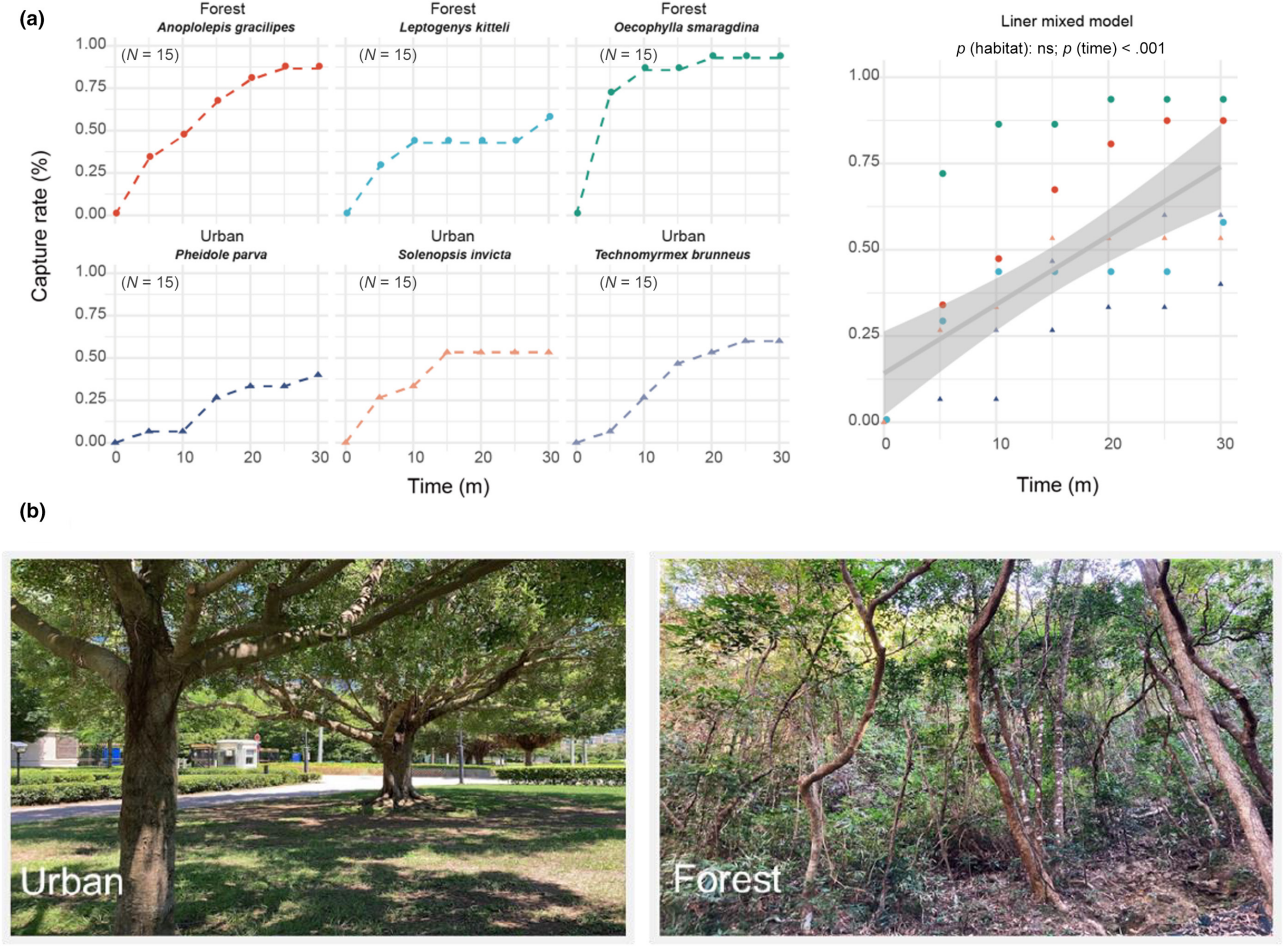
(a) In situ experiments exposing heat‐comatose ants to potential predators under natural conditions. Left: Species‐specific capture rate of heat‐comatose ants (six species) with exposure time in forest (top) and urban (bottom) habitats. Right: relationship between capture rate of heat‐comatose ants and exposure time when species were pooled together. The line is the linear regression, and the grey area represents the 95% confidence interval. (b) Photos of urban and forest habitats where the in situ experiments were conducted.

Overall, more than half of the heat‐comatose ants were attacked and removed by other ants (65%) after the 30‐min observation period. At 10 min since the comatose individuals were exposed to potential predators, their survival probability decreased to an average of 60% and dropped further to 35% after 30 min (Figure 2). Linear mixed model showed that the capture rate was significantly affected by exposure time (*p*‐value < .001) but not habitat (*p*‐value = .161, Table 1). The capture rates in the urban habitat were, however, 28% lower on average than in the forest habitat.

**TABLE 1:** Linear mixed model testing the relationship between capture rate and exposure time for heat‐comatose ants (*N* = 49 individuals) in the field under potential predation. Species was treated as a random effect nested within habitat.

Linear mixed model					
	Estimate	SE	df	*t*	*p*‐Value
Intercept	0.228	0.086	10.685	2.653	**.018**
Exposure time	0.022	0.003	36.000	7.792	**<.001**
Habitat	−0.177	0.117	10.876	−1.502	.161
Time: Habitat	−0.004	0.004	36.000	−1.079	.287

*Note*: Marginal *R* ^2^ ($R_{m}^{2}$): 0.655, Conditional *R* ^2^ ($R_{c}^{2}$): 0.802, AIC: −27.187. Bold values denote statistical significance at *p*‐values <.05 level.

### 3.2 Post heat‐coma recovery measurements

**FIGURE 3:**
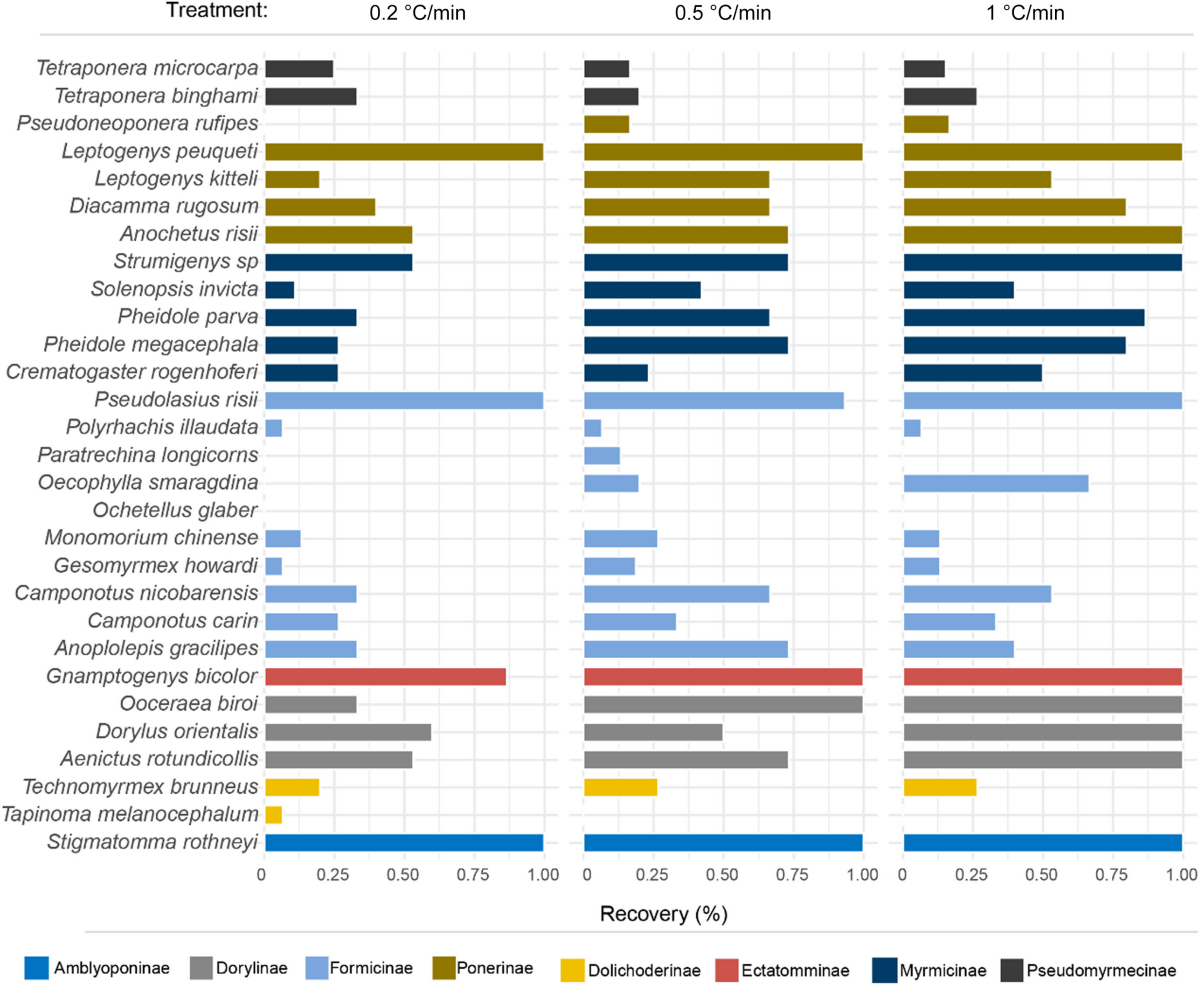
Recovery rate (%) after heat‐coma induced in dynamic thermal assays under three ramping rates across a total of 29 ant species from eight subfamilies (shown with different colors).

Most ant species tested could fully or partially recover after heat‐coma and resume locomotion ability, but some species such as *Ochetellus glaber* were unable to recover (i.e., recovery rate = 0%, Figure 3). The 10‐min recovery period was sufficient to allow most species (average = 88.4% across the three ramping treatments) to recover from the heat‐coma (Figure 3), with the average recovery rate increasing significantly from 32% to 54% when the ramping rate increased (*p‐*value < .001, Table 3). *Pseudoneoponera rufipes* and *Oecophylla smaragdina* failed to recover under the slowest ramping rate 0.2 °C/min while *Paratrechina longicornis* failed to recover under both the slowest and the fastest ramping rates. No phylogenetic signal was detected in the post heat‐coma recovery ability among all ramping rate treatments, based on Pagel's 𝜆 (*p*‐value > .05, Table 2) and Blomberg's *K* (*p*‐value > .05, Table 2) using a genus‐level phylogeny tree and genus‐averaged recovery rates.

**TABLE 2:** Phylogenetic signals of post heat‐coma recovery ability among the 29 assayed ant species.

	Treatment		
	0.2 °C/min	0.5 °C/min	1.0 °C/min
Pagel's 𝜆	4.5 e^−5^	0.079	0.404
*p*‐Value	1	.912	.370
Blomberg's *K*	0.679	0.740	0.790
*p*‐Value	.584	.276	.184

### 3.3 Post heat‐coma recovery and body mass

**FIGURE 4:**
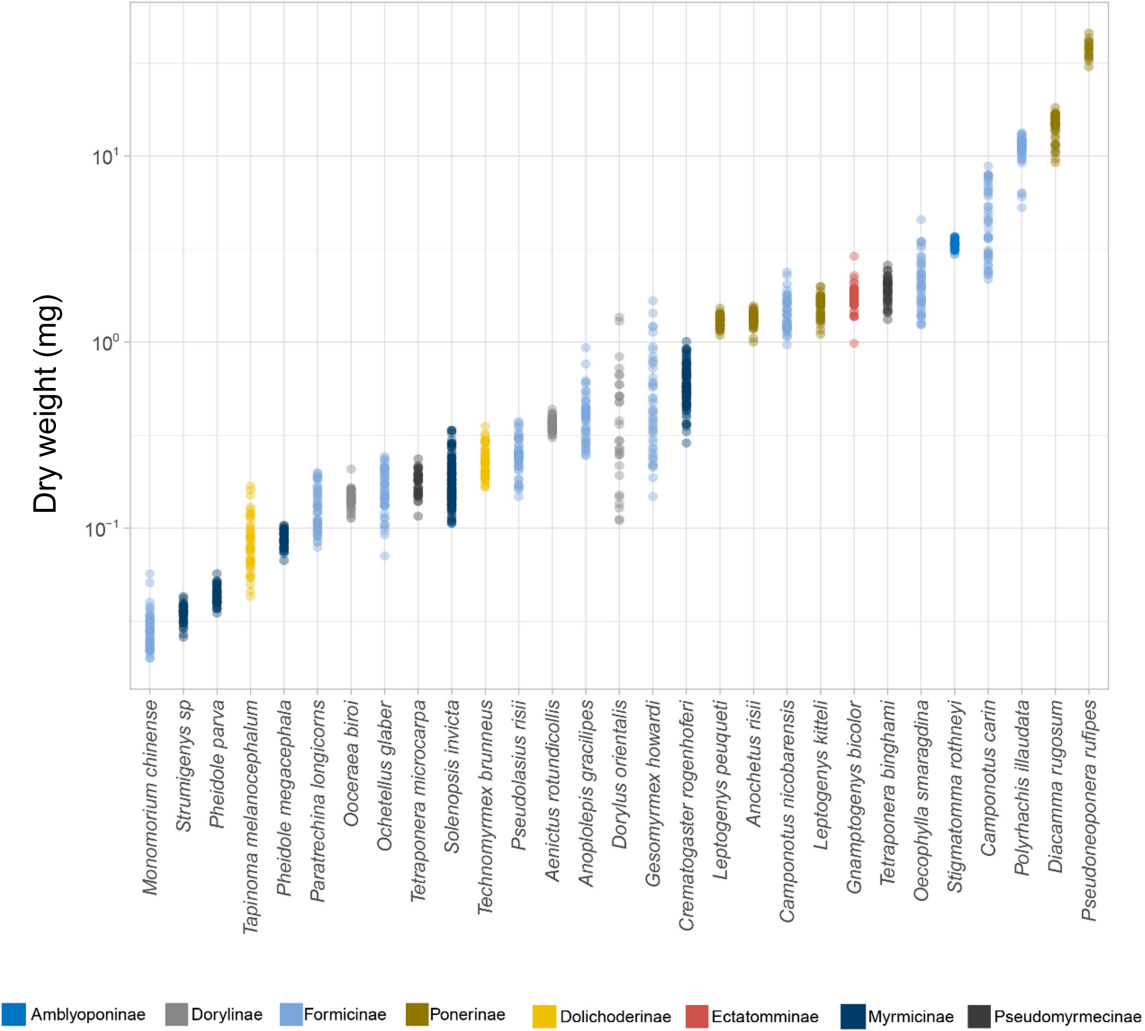
The body mass for 29 species from eight subfamilies (shown with different colors) with log‐transformation to illustrate the interspecific body mass variations.

**FIGURE 5:**
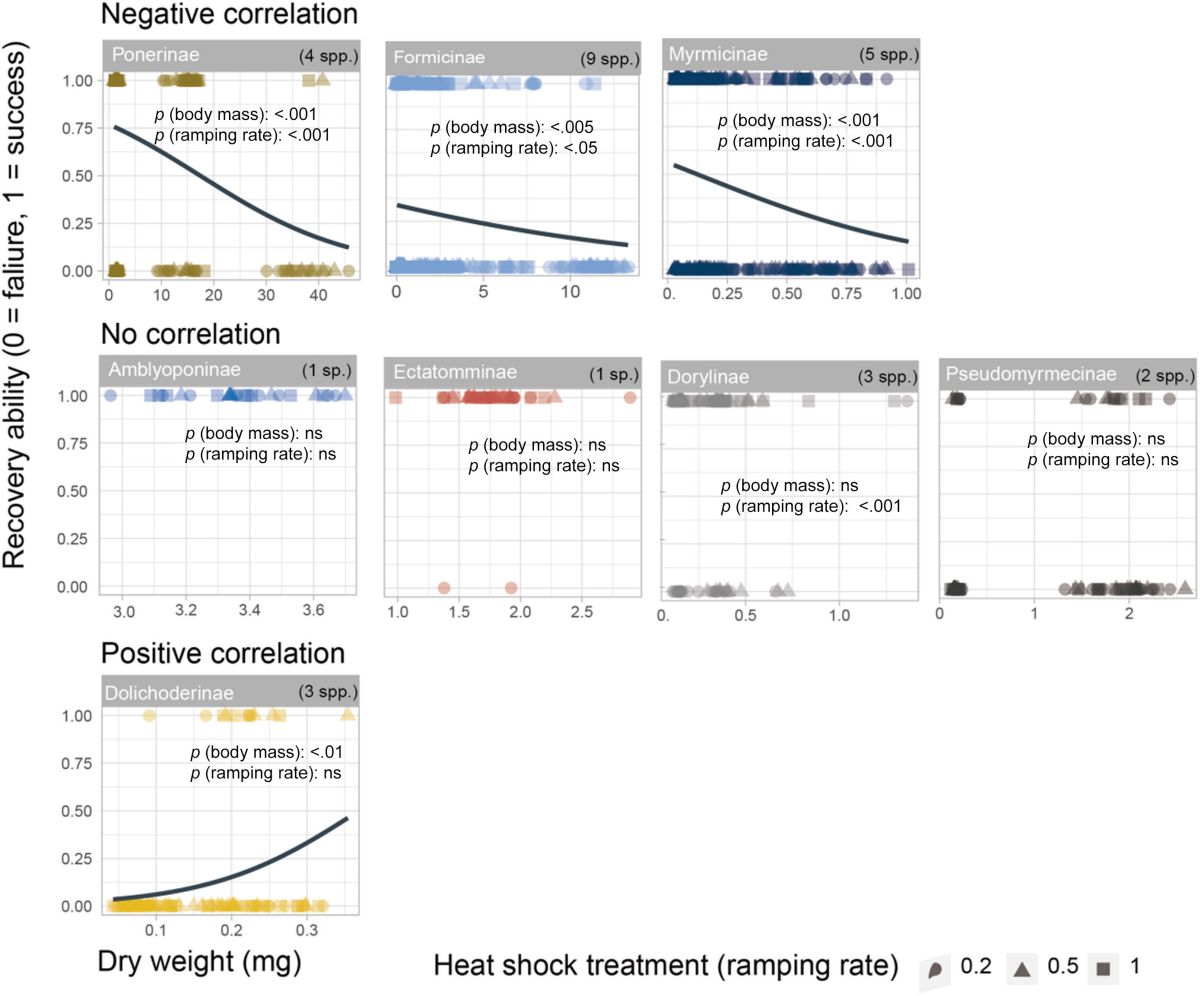
Generalized linear models investigating how post heat–coma recovery varied between heat shock treatments (ramping rates) and body mass of ants for each subfamily. The recovery failure (0) or success (1) was treated as the response variable whereas the ramping rate and body mass were the predictor variables. The *p*‐values for predictor variables, that is, body mass and ramping rate are shown for each subfamily (see: Table 3). ns, non–significance (*p‐*value > .05).

Recovery failure/success was significantly affected by the body mass, ramping rate, and their interaction for the eight subfamilies (Table 3). Body masses of the ant species tested varied 1297‐fold from 0.0289 mg in *Monomorium chinense* to 37.3830 mg in *Pseudoneoponera rufipes* (Figure 4). When all subfamilies were pooled for the analysis, all three predictor variables (i.e., ramping rate treatments, body mass, and the species identity) and their interactions affected recovery ability significantly (*p*‐values < .001, Table 3). In particular, recovery ability increased in smaller species (*p*‐value < .001). When the GzLM model was performed within each subfamily, recovery decreased with body mass in three subfamilies (i.e., Formicinae, Myrmicinae, and Ponerinae, *p*‐values < .001, Table 4) but increased with body mass in Dolichoderinae (Figure 5). Although a higher recovery rate was observed in higher ramping rates (Figure 3), recovery increased with ramping rates in four subfamilies only (*p*‐value < .05) and was independent of ramping rates in another four subfamilies (i.e., Amblyoponinae, Dolichoderinae, Ectatomminae, Pseudomyrmicinae), which had lower species diversities in the study sites and limited species were collected and accounted for in the GzLMs (Table 4).

**TABLE 3:** Analysis of deviance for the generalized linear model investigating variations in recovery rate with dry weight, heat‐coma treatment (i.e., ramping rates), and ant subfamily identify as the predictor variables for 29 species from eight subfamilies (*N* = 1373).

	LR Chisq	df	*p*‐Value
Body mass (DW)	35.72	1	**<.001**
Ramping rate	60.64	2	**<.001**
Subfamily	318.61	7	**<.001**
Body mass (DW) × Subfamily	24.71	7	**<.001**
Ramping rate × Subfamily	39.17	14	**<.001**
AIC = 1518.4			

*Note*: Bold values denote statistical significance at *p*‐values <.05 level.

Abbreviation: DW, Dry weight.

**TABLE 4:** Analysis of deviance for the generalized linear models investigating variations in recovery rates, with heat‐coma treatment (i.e., ramping rates) and dry weight as the predictor variables for each ant subfamily (eight in total).

	LR Chisq	df	*p*‐Value
Amblyoponinae (1 sp.)			
Body mass (DW)	0	1	1
Ramping rate	0	2	1
Body mass (DW) × Ramping rate	0	2	1
AIC: 12.0194			
Dolichoderinae (3 spp.)			
Body mass (DW)	6.8159	1	**<.01**
Ramping rate	0.0150	2	.9792
Body mass (DW) × Ramping rate	2.7093	2	.2580
AIC: 73.1238			
Dorylinae (3 spp.)			
Body mass (DW)	0.775	1	.3787
Ramping rate	37.426	2	**<.001**
Body mass (DW) × Ramping rate	9.523	2	**<.01**
AIC: 99.714			
Ectatomminae (1 sp.)			
Body mass (DW)	0.5221	1	.4699
Ramping rate	4.6937	2	.0956
Body mass (DW) × Ramping rate	0.0026	2	.9986
AIC:23.2751			
Formicinae (9 spp.)			
Body mass (DW)	7.9953	1	**<.005**
Ramping rate	7.0229	2	**<.05**
Body mass (DW) × Ramping rate	0.8170	2	.6645
AIC: 546.4953			
Myrmicinae (5 spp.)			
Body mass (DW)	16.822	1	**<.001**
Ramping rate	33.108	2	**<.001**
Body mass (DW) × Ramping rate	6.628	2	**<.05**
AIC: 450.3734			
Ponerinae (4 spp.)			
Body mass (DW)	27.1184	1	**<.001**
Ramping rate	16.5513	2	**<.001**
Body mass (DW) × Ramping rate	0.2497	2	.8826
AIC: 225.997			
Pseudomyrmecinae (2 spp.)			
Body mass (DW)	0.3863	1	.5343
Ramping rate	0.9946	2	.6082
Body mass (DW) × Ramping rate	0.5972	2	.7418
AIC: 98.8012			

*Note*: Bold values denote statistical significance at *p*‐values <.05 level.

Abbreviation: DW, Dry weight.

## 4 DISCUSSION

Numerous studies have shown that ectotherms are impacted by decreasing body size in response to novel, often warmer, environmental conditions (Lee et al., 2021; Tseng et al., 2018; Wu et al., 2019). Here we found that post heat‐coma recovery ability is an important ecophysiological trait to avoid ecological death and is negatively correlated with body size (Figure 1). Our results support the hypothesis that thermal resilience is higher in smaller ant species, which have a stronger recovery response to heat‐coma, than larger species. These results can be explained by biophysical laws where a small object has a lower thermal inertia and thus a faster heating/cooling rate as compared to larger objects made up of the same material (Planinšič & Vollmer, 2008). Therefore, in smaller individuals with lower masses and thus lower thermal inertia, their body temperatures change rapidly with the environment (Campbell & Norman, 1998). For instance, under natural and experimental conditions, body temperatures of small animals such as ants can heat up at a rate of over 1°C per second under sun exposure (Liao et al., 2019; Spicer et al., 2017). Similarly, smaller species can cool down faster due to a larger surface‐to‐volume ratio than larger species (Planinšič & Vollmer, 2008), and thus are more likely to recover as compared to larger species when returned to benign conditions after short‐term heat‐coma. In terms of heat exchange, convection plays an important role between the animal bodies and surrounding air. As a result, the surface area of an individual, which determines the rate of convection, would also control the rate of change in an individual's body temperature (Campbell & Norman, 1998; Kühsel et al., 2017).

This inverse relationship between thermal resilience and body mass in ants provides a physiological mechanistic explanation for the observation that ant communities have been increasingly composed of smaller‐sized species in hotter environments (Gibb et al., 2018; Lee et al., 2021; Nooten et al., 2019), a phenomenon also occurring for other taxa such as fishes, herptiles, and other ectotherms (e.g., Audzijonyte et al., 2020; Lee et al., 2021). Moreover, within the three dominant and species‐rich ant subfamilies, Myrmicinae, Formicinae, and Ponerinae, we also found that thermal resilience decreases with body mass among individuals with different sizes (Figure 5 and Table 3). These three subfamilies not only represent high biomass globally (Del Toro et al., 2012; Hölldobler & Wilson, 1990) but also constitute the majority of ant species diversity (82.4% overall ant diversity; 11,622/14,109; AntCat.org, 2022) and, therefore, play a critical role in global terrestrial ecosystems (Del Toro et al., 2012; Wilson, 1987). As large ants play a disproportionately important role in a number of ecosystem functions (Nooten et al., 2022; Woodward et al., 2005), the low or lack of thermal recovery ability in large individuals within these subfamilies are expected to bring cascading effects on ecosystems when these large, sometimes dominant species are impacted by the more frequent extreme events under climate warming.

Indeed, environmental temperatures can exceed upper thermal limits and induce heat‐coma for ants and other organisms (Kaspari, 2019). For instance, the upper thermal limit of *Polyrhachis illaudata* foraging in arboreal and ground strata, is 42–46°C (Leong et al., 2022) while strata surface temperature can reach 47°C during extremely hot days and induce heat‐coma in *P. illaudata* in situ (Leong C.M. and Tang E. personal observations Appendix S1: Figure S1). Forest environments, however, present a complex matrix of highly variable temperatures, with numerous thermal refuges allowing individuals to recover particularly in the closed, vegetated habitats (Lembrechts & Nijs, 2020; Zellweger et al., 2020). For example, temperatures recorded on the underside of leaves can be 15°C lower than their topside surface (Kaspari, 2019), and shaded, ground areas are significantly cooler than sun‐exposed canopy (Scheffers et al., 2014; Spicer et al., 2017).

In thermal refugia without extreme temperature conditions, however, predation and species interaction are expectedly more intense and comatose individuals with low recovery ability therefore are especially vulnerable. In our study, 40% of the comatose individuals were removed by other predatory ants within 10 min after their placement on the ground. Following their transport to the predatory ant colonies, the fate of these preys (comatose individuals) will be death as they would be consumed mainly as a protein food source for the larvae (Dussutour & Simpson, 2009). The ability to recover after heat‐coma is, therefore, ecologically relevant when individuals experiencing heat‐coma in hot, exposed canopy fall onto the ground or on other thermal refugia where temperatures are cooler but where, at the same time, predation may also be more intense. In our analysis, the predation risk was positively correlated with exposure time but independent of the habitat type (Figure 2), suggesting that recovery time after heat‐coma is vital for an individual's survival. The combination of heat‐coma and post heat‐coma recovery is thus critical for individual survival to avoid prolonged heat stress and minimize predation risk.

Although understanding thermal resilience is important to identify environmental filters in ecosystems, limited attention has been paid to post heat–coma recovery and the methods used to investigate this trait (Appendix S1: Table S1). The heat‐coma treatments (i.e., ramping rates) could affect post heat‐coma recovery measurements. Indeed, our results showed that species presented higher recovery when faster ramping rates were used (Table 3 and Figure 3), but the mechanisms behind this remain to this point unclear and should be further studied. To our knowledge, this is the first study focusing on the relationship between thermal resilience and body size in ectotherms. Despite recovery after heat‐coma entails substantial advantage to individual survival, one possible reason for the lack of study on such thermal resilience could be the difficulty for direct observation of recovery in the field, particularly in areas with high predation pressure where comatose individuals would be consumed rapidly (see Figure 1). In our predation experiment, surprisingly, all heat‐comatose individuals were preyed by predatory ants, indicating the severe ecological consequences of even temporary impairment in locomotion ability (Cerdá et al., 1998).

Under global climate change, average environmental temperature and the frequency of extremely hot days are predicted to increase (Lee et al., 2021; Zellweger et al., 2020), and as a consequence organisms are predicted to be increasingly exposed to extreme temperatures exceeding their upper thermal limits (Kellermann & van Heerwaarden, 2019; Shi et al., 2015). Our field observations in urban and coastal habitats, as well as previous studies (e.g., Schumacher & Whitford, 1974) in desert habitats, have shown that in sun‐exposed areas, extreme environmental temperatures >60°C, which are lethal to the majority of ectotherms, are possible. In our study, surface temperature could already reach 40 and 54°C on forest and on urban park grounds, respectively. Species encountering heat exhaustion or heat‐coma should therefore be more frequent under global change scenarios. Therefore, understanding how thermal resilience of species is determined by their ability to recover after heat‐coma is important in realistically predicting species performance in the future. We further identified species body size as a fundamental driver of their recovery ability after heat stress. As variations in species body size can also affect food webs through both top‐down and bottom‐up controls (Woodward et al., 2005), the variation in the recovery ability of species with different sizes are expected to induce cascading impacts on the ecosystems and their functions (Tan et al., 2021; Woodward et al., 2005). In conclusion, our study illustrates how post heat‐coma recovery could be used as an ecologically relevant trait in ectotherms and highlights the importance of body size in species responses to heat‐coma recovery. Such findings provide a mechanistic basis to predict how climatic changes shape animal communities and their size structures, as well as to determine species thermal resilience under future climate change scenarios.

## AUTHOR CONTRIBUTIONS

**Chi Man Leong:** Conceptualization (lead); data curation (lead); formal analysis (equal); investigation (lead); methodology (equal); validation (equal); visualization (equal); writing – original draft (equal); writing – review and editing (equal). **Tin Yan Hui:** Conceptualization (equal); formal analysis (equal); investigation (equal); methodology (equal); validation (equal); writing – original draft (equal); writing – review and editing (equal). **Benoit Guénard:** Conceptualization (lead); funding acquisition (equal); investigation (equal); methodology (equal); project administration (equal); supervision (equal); validation (equal); writing – original draft (equal); writing – review and editing (equal).

## CONFLICT OF INTEREST STATEMENT

There is no conflict of interest.

## Supporting information


Appendix S1

## Data Availability

Data are available within the article or its Supporting Information. Additionally, the raw data utilized in the statistical models are available on the Dryad data repository at https://doi.org/10.5061/dryad.qnk98sfnk.

## References

[ece310218-bib-0001] Angilletta, M. J., Jr. (2009). Thermal adaptation: A theoretical and empirical synthesis. Oxford University Press.

[ece310218-bib-0002] Angilletta, M. J. , Niewiarowski, P. H. , & Navas, C. A. (2002). The evolution of thermal physiology in ectotherms. Journal of Thermal Biology, 27, 249–268. 10.1016/S0306-4565(01)00094-8

[ece310218-bib-0003] AntCat . (2022). An online catalog of the ants of the world . https://antcat.org

[ece310218-bib-0004] Audzijonyte, A. , Richards, S. A. , Stuart‐Smith, R. D. , Pecl, G. , Edgar, G. J. , Barrett, N. S. , Payne, N. , & Blanchard, J. L. (2020). Fish body sizes change with temperature but not all species shrink with warming. Nature Ecology & Evolution, 4, 809–814. 10.1038/s41559-020-1171-0 32251381

[ece310218-bib-0005] Barton, K. , & Barton, M. K. (2015). Package ‘MuMin’ . Version, 1(18), 439.

[ece310218-bib-0006] Bar‐Ziv, M. A. , & Scharf, I. (2018). Thermal acclimation is not induced by habitat‐of‐origin, maintenance temperature, or acute exposure to low or high temperatures in a pit‐building wormlion (*Vermileo* sp.). Journal of Thermal Biology, 74, 181–186. 10.1016/j.jtherbio.2018.03.024 29801625

[ece310218-bib-0007] Bergmann, C. (1848). Über die Verhältnisse der Wärmeökonomie der Thiere zu ihrer Grösse. Gottinger Studien, 3, 595–708.

[ece310218-bib-0008] Bozinovic, F. , Bastías, D. A. , Boher, F. , Clavijo‐Baquet, S. , Estay, S. A. , & Angilletta, M. J., Jr. (2011). The mean and variance of environmental temperature interact to determine physiological tolerance and fitness. Physiological and Biochemical Zoology, 84, 543–552. 10.1086/662551 22030847

[ece310218-bib-0009] Brassard, F. , Leong, C.‐M. , Chan, H.‐H. , & Guénard, B. (2021). High diversity in urban areas: How comprehensive sampling reveals high ant species richness within one of the most urbanized regions of the world. Insects, 13, 358. 10.3390/d13080358

[ece310218-bib-0070] Bujan, J. F. , & Kaspari, M. (2017). Nutrition modifies critical thermal maximum of a dominant canopy ant. Journal of Insect Physiology, 102, 1–6. https://10.1016/j.jinsphys.2017.08.007 28830761 10.1016/j.jinsphys.2017.08.007

[ece310218-bib-0010] Bujan, J. , Yanoviak, S. P. , & Kaspari, M. (2016). Desiccation resistance in tropical insects: Causes and mechanisms underlying variability in a Panama ant community. Ecology and Evolution, 6, 6282–6291. 10.1002/ece3.2355 27648242 PMC5016648

[ece310218-bib-0011] Campbell, G. S. , & Norman, J. (1998). An introduction to environmental biophysics. Springer Science & Business Media.

[ece310218-bib-0012] Cerdá, X. , Retana, J. , & Cros, S. (1998). Critical thermal limits in Mediterranean ant species: Trade‐off between mortality risk and foraging performance. Functional Ecology, 12, 45–55. 10.1046/j.1365-2435.1998.00160.x

[ece310218-bib-0013] Cribari‐Neto, F. , & Zeileis, A. (2009). Beta regression in R . 34, 1–24.

[ece310218-bib-0014] Del Toro, I. , Ribbons, R. R. , & Pelini, S. L. J. M. N. (2012). The little things that run the world revisited: A review of ant‐mediated ecosystem services and disservices (Hymenoptera: Formicidae). Myrmecological News, 17, 133–146.

[ece310218-bib-0015] Dussutour, A. , & Simpson, S. J. (2009). Communal nutrition in ants. Current Biology, 19, 740–744. 10.1016/j.cub.2009.03.015 19345104

[ece310218-bib-0016] Economo, E. P. , Narula, N. , Friedman, N. R. , Weiser, M. D. , & Guénard, B. (2018). Macroecology and macroevolution of the latitudinal diversity gradient in ants. Nature Communications, 9, 1778. 10.1038/s41467-018-04218-4 PMC5934361 29725049

[ece310218-bib-0017] Eggleton, P. (2020). The state of the World's insects. Annual Review of Environment and Resources, 45, 61–82. 10.1146/annurev-environ-012420-050035

[ece310218-bib-0018] Furuki, T. , Umamoto, N. , Ohoka, W. , Nakajo, M. , Katagiri, C. , Wouthuyzen, S. , & Harada, T. (2017). Relationship between cool coma and heat coma temperatures in the oceanic sea skaters Halobates collected near the Sumatra Island in the Indian Ocean. Journal of Natural Sciences, 9, 145–157. 10.4236/ns.2017.95016

[ece310218-bib-0019] Gaitán‐Espitia, J. D. , Belén Arias, M. , Lardies, M. A. , & Nespolo, R. F. (2013). Variation in thermal sensitivity and thermal tolerances in an invasive species across a climatic gradient: Lessons from the land snail *Cornu aspersum* . PLoS One, 8, e70662. 10.1371/journal.pone.0070662 23940617 PMC3734266

[ece310218-bib-0020] Gao, J. , Zhang, W. , Dang, W. , Mou, Y. , Gao, Y. , Sun, B.‐J. , & Du, W.‐G. (2014). Heat shock protein expression enhances heat tolerance of reptile embryos. Proceedings of the Royal Society. Biological sciences, 281, 20141135. 10.1098/rspb.2014.1135 25080340 PMC4132679

[ece310218-bib-0021] García‐Robledo, C. , Kuprewicz, E. K. , Staines, C. L. , Erwin, T. L. , & Kress, W. J. (2016). Limited tolerance by insects to high temperatures across tropical elevational gradients and the implications of global warming for extinction. Proceedings of the National Academy of Sciences of the United States of America, 113, 680–685. 10.1073/pnas.1507681113 26729867 PMC4725502

[ece310218-bib-0022] Gibb, H. , Sanders, N. , Dunn, R. , Arnan, X. , Vasconcelos, H. , Donoso, D. , Andersen, A. , Silva, R. , Bishop, T. , & Gomez, C. (2018). Habitat disturbance selects against both small and large species across varying climates. Ecography, 41, 1184–1193. 10.1111/ecog.03244

[ece310218-bib-0023] González‐Tokman, D. , Córdoba‐Aguilar, A. , Dáttilo, W. , Lira‐Noriega, A. , Sánchez‐Guillén, R. A. , & Villalobos, F. (2020). Insect responses to heat: Physiological mechanisms, evolution and ecological implications in a warming world. Biological Reviews, 95, 802–821. 10.1111/brv.12588 32035015

[ece310218-bib-0024] Hoffmann, A. A. , Dagher, H. , Hercus, M. , & Berrigan, D. (1997). Comparing different measures of heat resistance in selected lines of *Drosophila melanogaster* . Journal of Insect Physiology, 43, 393–405. 12769901 10.1016/s0022-1910(96)00108-4

[ece310218-bib-0025] Hölldobler, B. , & Wilson, E. O. (1990). The ants. Belknap Press of Harvard University Press.

[ece310218-bib-0026] Johnson, D. J. , & Stahlschmidt, Z. R. (2020). City limits: Heat tolerance is influenced by body size and hydration state in an urban ant community. Ecology and Evolution, 10, 4944–4955. 10.1002/ece3.6247 32551072 PMC7297767

[ece310218-bib-0027] Jørgensen, L. B. , Robertson, R. M. , & Overgaard, J. (2020). Neural dysfunction correlates with heat coma and CTmax in *Drosophila* but does not set the boundaries for heat stress survival. The Journal of Experimental Biology, 223, jeb.218750. 10.1242/jeb.218750 32434804

[ece310218-bib-0028] Kaspari, M. (2019). In a globally warming world, insects act locally to manipulate their own microclimate. Proceedings of the National Academy of Sciences of the United States of America, 113, 5220–5222. 10.1073/pnas.1901972116 PMC6431203 30842283

[ece310218-bib-0029] Kaspari, M. , Clay, N. A. , Lucas, J. , Yanoviak, S. P. , & Kay, A. (2015). Thermal adaptation generates a diversity of thermal limits in a rainforest ant community. Global Change Biology, 21, 1092–1102. 10.1111/gcb.12750 25242246

[ece310218-bib-0030] Kellermann, V. , & van Heerwaarden, B. (2019). Terrestrial insects and climate change: Adaptive responses in key traits. Physiological Entomology, 44, 99–115. 10.1111/phen.12282

[ece310218-bib-0031] Kühsel, S. , Brückner, A. , Schmelzle, S. , Heethoff, M. , & Blüthgen, N. (2017). Surface area–volume ratios in insects. Insect Sci., 24, 829–841. 10.1111/1744-7917.12362 27234132

[ece310218-bib-0032] Lee, R. H. , Morgan, B. , Liu, C. , Fellowes, J. R. , & Guénard, B. (2021). Secondary forest succession buffers extreme temperature impacts on subtropical Asian ants. Ecological Monographs, 91, e1480. 10.1002/ecm.1480

[ece310218-bib-0033] Lembrechts, J. J. , & Nijs, I. (2020). Microclimate shifts in a dynamic world. Science, 368, 711–712. 10.1126/science.abc1245 32409462

[ece310218-bib-0034] Leong, C. M. , Shiao, S. F. , & Guénard, B. (2017). Ants in the city, a preliminary checklist of Formicidae (Hymenoptera) in Macau, one of the most heavily urbanized regions of the world. Asian Myrmecology, 9, 1–20. 10.20362/am.009014

[ece310218-bib-0035] Leong, C. M. , Tsang, T. P. N. , & Guénard, B. (2022). Testing the reliability and ecological implications of ramping rates in the measurement of critical thermal maximum. PLoS One, 17, e0265361. 10.1371/journal.pone.0265361 35286353 PMC8920270

[ece310218-bib-0036] Liao, H. , Du, T. , Zhang, Y. , Shi, L. , Huai, X. , Zhou, C. , & Deng, J. (2019). Capacity for heat absorption by the wings of the butterfly *Tirumala limniace* (Cramer). PeerJ, 7, e6648. 10.7717/peerj.6648 30941273 PMC6438159

[ece310218-bib-0037] Lucky, A. , Trautwein, M. D. , Guénard, B. S. , Weiser, M. D. , & Dunn, R. R. (2013). Tracing the rise of ants – Out of the ground. PLoS One, 8, e84012. 10.1371/journal.pone.0084012 24386323 PMC3873401

[ece310218-bib-0038] Lutterschmidt, W. I. , & Hutchison, V. H. (1997a). The critical thermal maximum: Data to support the onset of spasms as the definitive end point. Canadian Journal of Zoology, 75, 1553–1560. 10.1139/z97-782

[ece310218-bib-0039] Lutterschmidt, W. I. , & Hutchison, V. H. (1997b). The critical thermal maximum: History and critique. Canadian Journal of Zoology, 75, 1561–1574. 10.1139/z97-783

[ece310218-bib-0040] Ma, C.‐S. , Ma, G. , & Pincebourde, S. (2020). Survive a warming climate: Insect responses to extreme high temperatures. Annual Review of Entomology, 66, 163–184. 10.1146/annurev-ento-041520-074454 32870704

[ece310218-bib-0041] Malcolm, J. R. , Liu, C. , Neilson, R. P. , Hansen, L. , & Hannah, L. E. E. (2006). Global warming and extinctions of endemic species from biodiversity hotspots. Conservation Biology, 20, 538–548. 10.1111/j.1523-1739.2006.00364.x 16903114

[ece310218-bib-0042] Marshall, D. J. , Dong, Y.‐W. , McQuaid, C. D. , & Williams, G. A. (2011). Thermal adaptation in the intertidal snail *Echinolittorina malaccana* contradicts current theory by revealing the crucial roles of resting metabolism. The Journal of Experimental Biology, 214, 3649–3657. 10.1242/jeb.059899 21993794

[ece310218-bib-0043] McMahon, R. F. (1976). Effluent‐induced interpopulation variation in the thermal tolerance of Physa virgata Gould. Comparative Biochemistry Physiology Part A: Physiology, 55, 23–28. 10.1016/0300-9629(76)90117-1 8240

[ece310218-bib-0044] Mori, N. , & Kimura, M. T. (2008). Selection for rapid and slow recovery from chill‐ and heat‐coma in *Drosophila melanogaster* . Biological Journal of the Linnean Society, 95, 72–80. 10.1111/j.1095-8312.2008.01041.x

[ece310218-bib-0045] Mueller, B. , & Seneviratne, S. I. (2012). Hot days induced by precipitation deficits at the global scale. Proceedings of the National Academy of Sciences of the United States of America, 109, 12398–12403. 10.1073/pnas.1204330109 22802672 PMC3411978

[ece310218-bib-0046] Nooten, S. , Chan, K. H. , Schultheiss, P. , Bogar, T. A. , & Guénard, B. (2022). Ant body size mediates functional performance and species interactions in carrion decomposer communities. Functional Ecology, 36, 1279–1291. 10.1111/1365-2435.14039

[ece310218-bib-0047] Nooten, S. , Schultheiss, P. , Rowe, R. C. , Facey, S. L. , & Cook, J. M. (2019). Habitat complexity affects functional traits and diversity of ant assemblages in urban green spaces (Hymenoptera: Formicidae). Myrmecological News, 29, 67–77. 10.25849/myrmecol.news_029:067

[ece310218-bib-0048] Pinsky, M. L. , Eikeset, A. M. , McCauley, D. J. , Payne, J. L. , & Sunday, J. M. (2019). Greater vulnerability to warming of marine versus terrestrial ectotherms. Nature, 569, 108–111. 10.1038/s41586-019-1132-4 31019302

[ece310218-bib-0049] Planinšič, G. , & Vollmer, M. (2008). The surface‐to‐volume ratio in thermal physics: From cheese cube physics to animal metabolism. European Journal of Physics, 29, 369–384. 10.1088/0143-0807/29/2/017

[ece310218-bib-0050] R Development Core Team . (2020). R: A language and environment for statistical computing. R Foundation for Statistical Computing.

[ece310218-bib-0051] Rahmstorf, S. , & Coumou, D. (2011). Increase of extreme events in a warming world. Proceedings of the National Academy of Sciences of the United States of America, 108, 17905–17909. 10.1073/pnas.1101766108 22025683 PMC3207670

[ece310218-bib-0071] Revell, L. J. (2012). phytools: an R package for phylogenetic comparative biology (and other things) . 3(2), 217–223. https://10.1111/j.2041‐210X.2011.00169.x

[ece310218-bib-0052] Roeder, K. A. , Roeder, D. V. , & Bujan, J. (2021). Ant thermal tolerance: A review of methods, hypotheses, and sources of variation. Annals of the Entomological Society of America, 114, 459–469. 10.1093/aesa/saab018

[ece310218-bib-0053] Rolán‐Alvarez, E. , Carvajal‐Rodríguez, A. , de Coo, A. , Cortés, B. , Estévez, D. , Ferreira, M. , González, R. , & Briscoe, A. D. J. E. (2015). The scale‐of‐choice effect and how estimates of assortative mating in the wild can be biased due to heterogeneous samples. Evolution, 69, 1845–1857. 26085130 10.1111/evo.12691

[ece310218-bib-0054] Scheffers, B. R. , Edwards, D. P. , Diesmos, A. , Williams, S. E. , & Evans, T. A. (2014). Microhabitats reduce animal's exposure to climate extremes. Global Change Biology, 20, 495–503. 10.1111/gcb.12439 24132984

[ece310218-bib-0055] Schumacher, A. , & Whitford, W. G. (1974). The foraging ecology of two species of Chihuahuan desert ants: *Formica perpilosa* and *Trachyrmyrmex smithi neomexicanus* (Hymenoptera Formicidae). Insectes Sociaux, 21(3), 317–330. 10.1007/BF02226923

[ece310218-bib-0056] Séguin, A. , Harvey, É. , Archambault, P. , Nozais, C. , & Gravel, D. (2014). Body size as a predictor of species loss effect on ecosystem functioning. Scientific Reports, 4, 4616. 10.1038/srep04616 24714619 PMC3980226

[ece310218-bib-0057] Shi, N. N. , Tsai, C.‐C. , Camino, F. , Bernard, G. D. , Yu, N. , & Wehner, R. (2015). Keeping cool: Enhanced optical reflection and radiative heat dissipation in Saharan silver ants. Science, 349, 298–301. 10.1126/science.aab3564 26089358

[ece310218-bib-0058] Sinclair, B. J. , Marshall, K. E. , Sewell, M. A. , Levesque, D. L. , Willett, C. S. , Slotsbo, S. , Dong, Y. , Harley, C. D. G. , Marshall, D. J. , Helmuth, B. S. , & Huey, R. B. (2016). Can we predict ectotherm responses to climate change using thermal performance curves and body temperatures? Ecology Letters, 19, 1372–1385. 10.1111/ele.12686 27667778

[ece310218-bib-0059] Spicer, M. E. , Stark, A. Y. , Adams, B. J. , Kneale, R. , Kaspari, M. , & Yanoviak, S. P. (2017). Thermal constraints on foraging of tropical canopy ants. Oecologia, 183, 1007–1017. 10.1007/s00442-017-3825-4 28132105

[ece310218-bib-0060] Tan, H. , Hirst, A. G. , Atkinson, D. , & Kratina, P. (2021). Body size and shape responses to warming and resource competition. Functional Ecology, 35, 1460–1469. 10.1111/1365-2435.13789

[ece310218-bib-0061] Tross, J. , Wolf, H. , & Pfeffer, S. E. (2021). Allometry in desert ant locomotion (*Cataglyphis albicans* and *Cataglyphis bicolor*) – Does body size matter? Journal of Experimental Biology, 224, jeb242842. 10.1242/jeb.242842 34477873

[ece310218-bib-0062] Tseng, M. , Kaur, K. M. , Soleimani Pari, S. , Sarai, K. , Chan, D. , Yao, C. H. , Porto, P. , Toor, A. , Toor, H. S. , & Fograscher, K. J. J. O. A. E. (2018). Decreases in beetle body size linked to climate change and warming temperatures. The Journal of Animal Ecology, 87, 647–659. 10.1111/1365-2656.12789 29380382

[ece310218-bib-0063] Wehner, R. , & Wehner, S. (2011). Parallel evolution of thermophilia: Daily and seasonal foraging patterns of heat‐adapted desert ants: *Cataglyphis* and *Ocymyrmex* species*. Physiological Entomology, 36, 271–281. 10.1111/j.1365-3032.2011.00795.x

[ece310218-bib-0064] Wickham, H. (2016). ggplot2: Elegant graphics for data analysis. Springer.

[ece310218-bib-0065] Willot, Q. , Loos, B. , & Terblanche, J. S. (2021). Interactions between developmental and adult acclimation have distinct consequences for heat tolerance and heat stress recovery. Journal of Experimental Biology, 224:jeb242479. 10.1242/jeb.242479 34308995

[ece310218-bib-0066] Wilson, E. O. (1987). The little things that run the world (the importance and conservation of invertebrates). Conservation Biology, 1, 344–346.

[ece310218-bib-0067] Woodward, G. , Ebenman, B. , Emmerson, M. , Montoya, J. M. , Olesen, J. M. , Valido, A. , & Warren, P. H. (2005). Body size in ecological networks. Trends in Ecology & Evolution, 20, 402–409. 10.1016/j.tree.2005.04.005 16701403

[ece310218-bib-0068] Wu, C. H. , Holloway, J. D. , Hill, J. K. , Thomas, C. D. , Chen, I. C. , & Ho, C. K. (2019). Reduced body sizes in climate‐impacted Borneo moth assemblages are primarily explained by range shifts. Nature Communications, 10, 4612. 10.1038/s41467-019-12655-y PMC6787050 31601806

[ece310218-bib-0069] Zellweger, F. , Frenne, P. D. , Lenoir, J. , Vangansbeke, P. , Verheyen, K. , Bernhardt‐Römermann, M. , Baeten, L. , Hédl, R. , Berki, I. , Brunet, J. , Calster, H. V. , Chudomelová, M. , Decocq, G. , Dirnböck, T. , Durak, T. , Heinken, T. , Jaroszewicz, B. , Kopecký, M. , Máliš, F. , … Coomes, D. (2020). Forest microclimate dynamics drive plant responses to warming. Science, 368, 772–775. 10.1126/science.aba6880 32409476

