# Supplementary material for: The role of body mass in limiting post heat‐coma recovery ability in terrestrial ectotherms
Source: Ecol Evol. 2023 Jun 23;13(6):e10218. doi: 10.1002/ece3.10218 (PMC10288262; doi:10.1002/ece3.10218)
Supplement: Supplementary file 1 — Appendix S1 [file ECE3-13-e10218-s001.docx]

**Supporting information:** The role of body mass in limiting post heat-coma recovery ability in terrestrial ectotherms

**Appendix S1. Recovery temperature design and microclimatic information**

Under the assumption that heat tolerant species usually live and lose muscle control under hotter thermal conditions (Angilletta Jr, 2009), we attempt to account for this and test it by setting the recovery temperature at a value of CT_max_ minus 10 °C, with each value being specific to the species tested. We then compare the recovery rate for specimens between this CT_max_ adjusted value (treatment group) and compare it with the room temperature (control group). Three species (*Pheidole parva, Technomyrmex brunneus,* and *Solenopsis invicta*) from open habitat and three species (*Gnamptogenys bicolor, Leptogenys kitteli,* and *Oecophylla smaragdina*) from closed habitat were tested with three treatments due to the ramping rate design. For each species and ramping rate treatment (0.2, 0.5, or 1.0 °C min^-1^), 20 individuals from different colonies were tested; 10 for control group (i.e., room temperature) and 10 for treatment group. Ant recovery rate after the heat-coma was tested between the control group and treatment group for six species, no significant differences were detected (see below table). Therefore, the room temperature as recovery treatment temperature was used for all 29 species in the post heat-coma experiments and measurements.

**Table S1.** Comparison between control temperature and treatment temperature for six species in terms of recovery success or failure after the heat-coma. Recovery rate is the proportion of recovered individuals over all tested individuals.

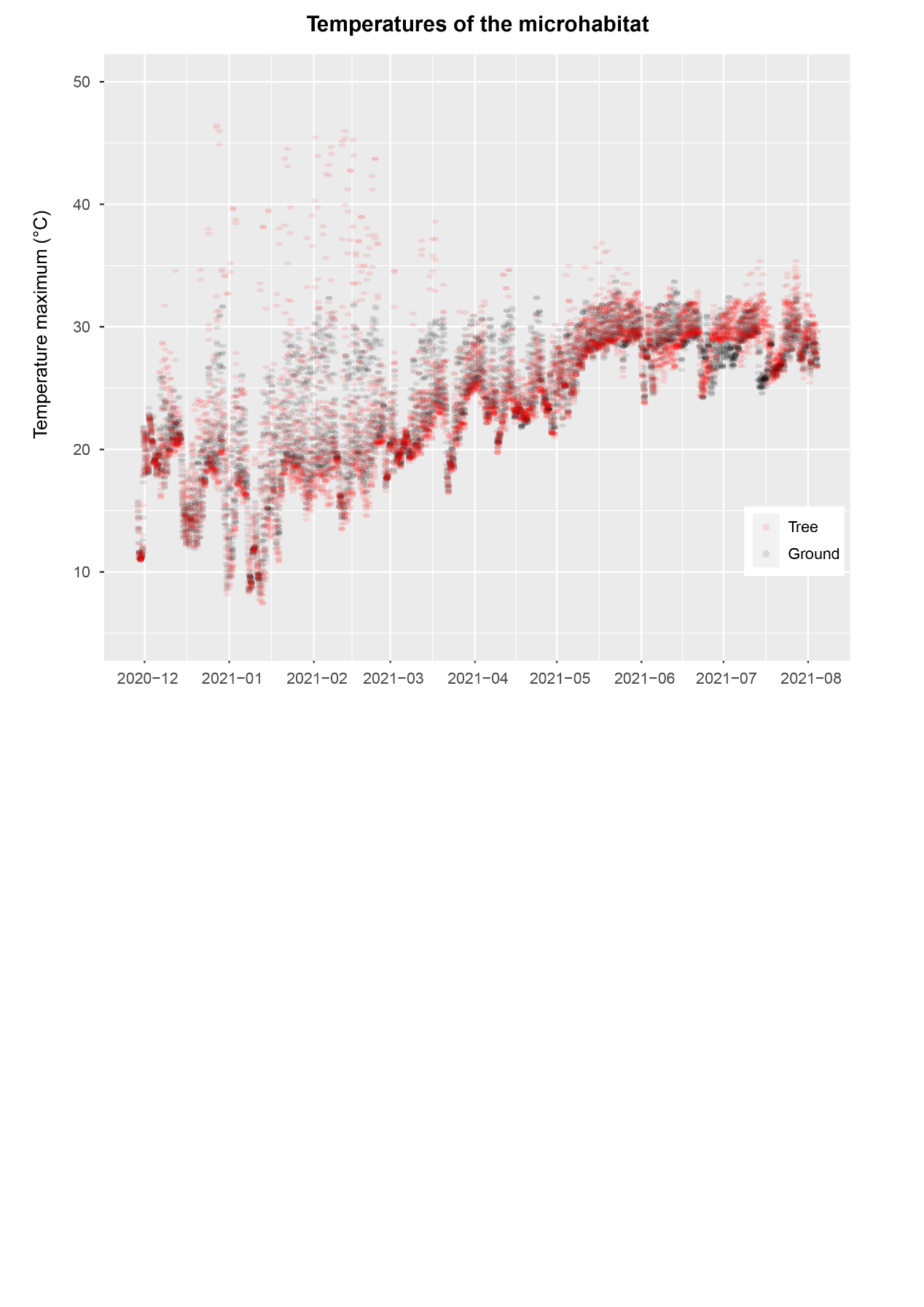


Figure S1. Microclimate data of tree and ground, where the studying ant species could be exposed which thermal conditions. Six dataloggers were set on trees two meters above the ground or ground surface equally. Temperature maximum (°C) is recorded hourly for nine months included the dry and wet seasons. Jan, Feb, Mar, and Dec = dry seasons; Apr, May, June, Jul, and Aug = wet seasons.

**Supporting information:** The role of body mass in limiting post heat-coma recovery ability in terrestrial ectotherms

**Appendix S2. Literature review of measurement methods of post heat-coma recovery**

Table S1. Literature review of recovery time after heat-coma till 2022. Abbreviation: temp. = temperature, hr = hour, NA = not applicable.

| **Investigated species** | **Heat treatment**  **to induce heat-coma** | **Recovery temp.** | **Observation for recovery** |
| --- | --- | --- | --- |
| **Fruit fly** (1 species)  (*Drosophila melanogaster*)  Ref.: (Mori & Kimura, 2008) | 38 °C  0.66 – 0.91 hr | 25 °C | Min: NA.  Max: 1 hr |
| **Fruit fly** (1 species)  (*Drosophila melanogaster*)  Ref.: (Bozinovic et al., 2011) | 38 °C  0.5 hr | 21 °C ± 1°C | Min: NA.  Max: 0.34 hr |
| **Fruit fly** (1 species)  (*Drosophila melanogaster*)  Ref.: (Willot, Loos & Terblanche, 2021) | 41 °C  6 hrs | 21 °C | Min: 0.3 hr  Max: 12.5 hrs |
| **Sea skater** (3 species)  *(Halobates germanus*, *H. micans*, *H. princeps)*  Ref.: (Furuki et al., 2017) | 0.20 – 0.34 °C / min | NA. | Min: NA.  Max: 2 hrs |
| **Snail** (1 species)  *(Echinolittorina malaccana)*  Ref.: (Marshall et al., 2011) | 48 or 50 °C  2, 4, 6, 8, or 10 hours | 25 °C | Min: 1 hrs  Max: 8 hrs |
| **Snail** (1 species)  (*Cornu aspersum*)  Ref.: (Gaitán-Espitia et al., 2013) | 35 or 39 °C  39 to 40 °C  40 to 45 °C  No specific time | 20 °C | Min: N.A.  Max: 0.45 hr |
| **Snail** (1 species)  (*Chlorostoma funebralis*)  Ref.: (Gleason & Burton, 2013) | 37, 38, 39, 40, and 41 °C  5.5 hrs for each temp. | NA. | Min: 0.3 hr  Max: 5.5 hrs |
| **Worm-lion** (1 species)  (Vermileo sp.)  Ref.: (Bar-Ziv & Scharf, 2018) | 48.0 ± 0.2 °C  for 3.5 min | 23 °C  26 °C  28 °C  31 °C | Min: NA.  Max: 1.67 hrs |

**References:**

Bar-Ziv, M.A. & Scharf, I. (2018) Thermal acclimation is not induced by habitat-of-origin, maintenance temperature, or acute exposure to low or high temperatures in a pit-building wormlion (*Vermileo* sp.). *Journal of Thermal Biology,* **74,** 181-186. <https://doi.org/10.1016/j.jtherbio.2018.03.024>

Bozinovic, F., Bastías, D.A., Boher, F., Clavijo-Baquet, S., Estay, S.A. & Angilletta, M.J., Jr. (2011) The mean and variance of environmental temperature interact to determine physiological tolerance and fitness. *Physiological and Biochemical Zoology,* **84,** 543-552. <https://doi.org/10.1086/662551>

Furuki, T., Umamoto, N., Ohoka, W., Nakajo, M., Katagiri, C., Wouthuyzen, S. & Harada, T. (2017) Relationship between cool coma and heat coma temperatures in the oceanic sea skaters Halobates collected near the Sumatra Island in the Indian Ocean. *Journal of Natural Sciences,* **9,** 145-157.

Gaitán-Espitia, J.D., Belén Arias, M., Lardies, M.A. & Nespolo, R.F. (2013) Variation in thermal sensitivity and thermal tolerances in an invasive species across a climatic gradient: lessons from the land snail *Cornu aspersum*. *PLoS One,* **8,** e70662. <https://doi.org/10.1371/journal.pone.0070662>

Gleason, L.U. & Burton, R.S. (2013) Phenotypic evidence for local adaptation to heat stress in the marine snail Chlorostoma (formerly Tegula) funebralis. *Journal of Experimental Marine Biology and Ecology,* **448,** 360-366. <https://doi.org/10.1016/j.jembe.2013.08.008>

Marshall, D.J., Dong, Y.-w., McQuaid, C.D. & Williams, G.A. (2011) Thermal adaptation in the intertidal snail *Echinolittorina malaccana* contradicts current theory by revealing the crucial roles of resting metabolism. *The Journal of Experimental Biology,* **214,** 3649-3657. <https://doi.org/10.1242/jeb.059899>

Mori, N. & Kimura, M.T. (2008) Selection for rapid and slow recovery from chill- and heat-coma in *Drosophila melanogaster*. *Biological Journal of the Linnean Society,* **95,** 72-80. <https://doi.org/10.1111/j.1095-8312.2008.01041.x>

Willot, Q., Loos, B. & Terblanche, J.S. (2021) Interactions between developmental and adult acclimation have distinct consequences for heat tolerance and heat stress recovery. *Journal of Experimental Biology,* **224**. <https://doi.org/10.1242/jeb.242479>
